# A Smart Design Strategy for Super‐Elastic Hydrogel with Long‐Term Moisture, Extreme Temperature Resistance, and Non‐Flammability

**DOI:** 10.1002/advs.202100320

**Published:** 2021-06-19

**Authors:** Haiquan Zhang, Zijing Liu, Junping Mai, Ning Wang, Houji Liu, Jie Zhong, Xianmin Mai

**Affiliations:** ^1^ State Key Laboratory of Marine Resource Utilization in South China Sea Hainan University Haikuo 570228 P. R. China; ^2^ School of Architecture Southwest Minzu University Chengdu 610041 P. R. China

**Keywords:** anti‐freezing and heating resistant, composite materials, long‐term moisture, non‐flammable materials, super‐elastic hydrogels

## Abstract

Elastic hydrogel is a promising material category for designing biological muscles, repairable building materials, flexible electronic devices, and vulcanized rubber substitutes, which is required to have a long life, good self‐healing performance and extreme temperature tolerance. Herein, a super‐elastic mineral hydrogel is developed with long‐lasting moisture, based on dynamic physical crosslinking between hydrated calcium ion clusters and amide groups of polyacrylamide (PAM). The complex hydrogel exhibits a super stretchability of 13 600% at room temperature, and can maintain the super flexibility in a wide temperature range of −40–50 °C or for a long period of 28 days. Particularly, the soft material cannot be ignited under an open flame at 400–500 °C, because of coupling dual flame retardant mechanisms containing the endothermic effect of liquid water evaporation and the barrier effect of calcium mineral salt on oxygen. In conclusion, the novel complex hydrogel with excellent tensile property, stability in extreme temperature or long operating time, and flame retardancy may become a promising candidate in the fields of agriculture, food, construction, medicine, and machinery.

## Graphical abstract

Dynamic physical crosslinking of calcium (II) with in situ polymerized polyacrylamide provides a new mechanism for stress/energy dispersion of soft materials during stretching. As‐prepared complex hydrogel exhibits stable mechanical performance for a long‐term or under extreme temperature, and has ideal flame‐retardant ability due to the water‐locking effect of calcium (II) and barrier effect on oxygen.

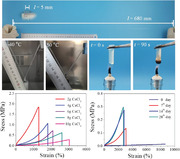

## 1 Introduction

The past decade has witnessed the rapid development of stretchable polymer hydrogels containing a large amount of water in their porous network structures, which have been widely applied in agriculture,^[^ ^1^ ^]^ food chemistry,^[^ ^2^ ^]^ artificial muscle,^[^ ^3^ ^]^ tissue scaffold,^[^ ^4^ ^]^ drug delivery carrier,^[^ ^5^ ^]^ and electronic component.^[^ ^6^ ^]^ In general, polymer hydrogels suffer from low mechanical strength, poor toughness, limited recoverability, and self‐healing property due to lack of fracture‐reformation dynamic equilibrium and/or efficient energy dissipation mechanisms.^[^ ^7^ ^]^

Many efforts have been contributed to develop tough hydrogels with new micro‐structures and toughening mechanisms through integrating hydrophobic association, ionically cross‐linked,DD including sliding‐ring hydrogel,^[^ ^8^ ^]^ double network hydrogel,^[^ ^9^ ^]^ nanocomposite hydrogel,^[^ ^10^ ^]^ macromolecular microsphere composite hydrogel,^[^ ^11^ ^]^ and hydrophobic association hydrogel (HAH).^[^ ^12^ ^]^ A physically linked Agar/PAM double network hydrogel exhibited a maximum strain of 5260%, corresponding to a fracture energy of 1000 J m^−2^.^[^ ^13^ ^]^ PAM chains were limited to the surface of polystyrene‐*co*‐poly (*N,N*‐dimethylacrylamide) microspheres by multiple hydrogen bonds, and corresponding HAH gel displayed a fracture strain of 3060% and a fracture stress of 1365 kPa.^[^ ^14^ ^]^

Most of the hydrogel materials currently studied are still difficult to maintain good mechanical performances in high or low temperature environments,^[^ ^15^ ^]^ because the weak hydrogen bond force with only 20–30 kJ mol^−1^ inevitably vaporizes or crystallizes liquid water. Note that the loss or freezing of the water causes the hydrogels to harden, which severely weakens the tensile, compression, and shear resistance properties. In addition, conventional hydrogels are gradually dehydrated after being ignited and burned into a small amount of ashes, resulting in limited real‐world applications. How to maintain these excellent comprehensive properties in a wide temperature window is a major challenge for designing high‐quality hydrogels with good stretchability, self‐healing, long‐term stability, anti‐freezing, heating resistant, and flame retardancy.

**Figure 1:**
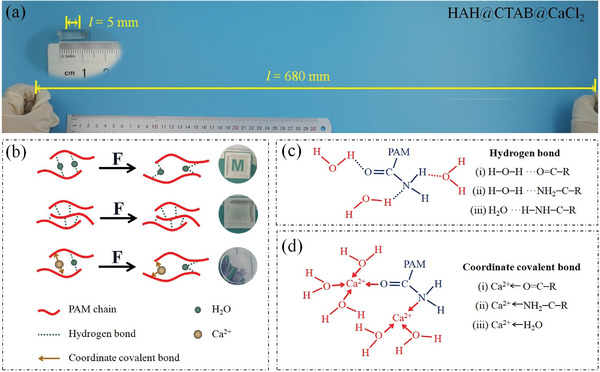
Design strategy for a multi‐functional super‐elastic hydrogel based on dynamic equilibrium hydrogen/coordination bonds. a) Exhibition of superior tensile property for the hydrogel with an *m*_PAM_/*m*_calcium chloride_ mass ratio of 5:10. b) Schematic illustration of the as‐prepared hydrogel undergoing reversible rupture and reconstruction during tensile stretching (using a force *F*). c) Hydrogen bonds of the PAM‐water. d) Coordination bonds formed by Ca‐ions with amide groups of PAM and hydroxyl of H_2_O.

Herein, we successfully achieved a novel hydrogel with water‐locking effect by elaborately designed dynamic physical crosslinking 3D structure, which simultaneously possesses super high stretchability (failure strain more than 13 600%), long‐term stability in a wide temperature window of −40–50 °C, and good flame retardancy (99.8% of limiting oxygen index, LOI). We believe the super‐elastic hydrogel can be used in civil construction to replace the easily aging/hardening rubber, improve the passive dimming and humidity control capabilities of curtain and window, anti‐cracking, and flame retardant properties of wood ecological building materials. As shown in **Figure** **1**, multiple modes of hydrogen/coordination bond breaking, exchanging and re‐formation offer energy dissipation mechanisms. [Ca(H_2_O)*_x_*]^2+^ (*x* = 1–6) hydrated ion cluster^[^ ^16^ ^]^ exhibits high bonding energy of 103.4–238.2 kJ mol^−1^, which disrupts the formation of crystal lattices of ice at low temperature and prevents the evaporation of water at high temperature. We believe that our novel design strategy could be generally applied to develop a new generation of tough hydrogel materials.

## 2 Results and Discussion

### 2.1 Design Principle of the HAH Hydrogel

Hydrogen bonds (20–30 kJ mol^−1^), derived from the amide groups (O═C─NH_2_) of PAM chain and water molecules, can be broken and re‐formed reversibly for energy dissipation on stretching and self‐healing on being damaged. However, traditional hydrogels cannot retain a long‐term wet state by virtue of weak intermolecular interactions, which results in the failure of super‐elasticity of hydrogels. We chose calcium chloride to improve the mechanical stability of hydrogels in a wide temperature window based on the following considerations, as shown in Figure 1. i) Mechanical performances: Ca metal ions still are held near the ligands by the stronger interactions (Ca ← CONH_2_) during stretching, which allow a rapid bond reconstruction. Based on the rapid energy and force dispersion mechanism, the designed hydrogel can be quickly stretched from 5 mm effective length to 680 mm. ii) Water retention performance: Ca‐ion is also easy to form coordination bond with hydroxyl (─OH) electron‐donating groups, thereby obtaining structurally stable aqueous ion clusters [Ca(H_2_O)*_x_*]^2+^ (*x* = 1–6). Calcium hydrate ions obviously enhance the water retention capacity of the as‐prepared hydrogel, and prevent PAM poly chains from losing super‐elastic properties. On the other hand, calcium ions reduce the bonding ability of hydrogen bonds between water molecules, the saturated vapor pressure and chemical potential (enthalpy of solution of CaCl_2_, −176.2cal g^−1^) of solvent water, and inhibit the formation and growth of crystal nuclei in the hydrogel network. These comprehensive factors can improve the anti‐freeze performance of the hydrogel. iii) Flame retardancy: Liquid water molecules locked by calcium ions can quickly evaporate and absorb a lot of heat when threatened by fire, while CaCl_2_ inorganic salt covers the surface of the water loss gel to avoid direct contact between flammable organic substances and oxygen. The dual protection mechanisms make the self‐synthesized hydrogel possess surprisingly flame retardant properties.

### 2.2 Mechanical Properties of the As‐Prepared Hydrogel

#### 2.2.1 Mechanical Performance

**Figure 2:**
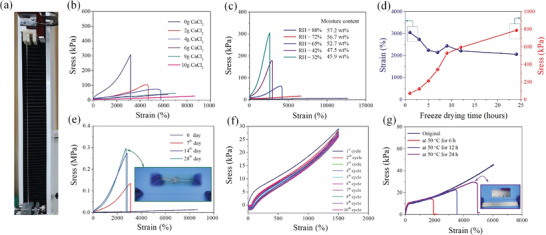
Mechanical properties of the complex hydrogel. a) Representative photograph of tensile testing. b) Tensile stress curves of the fresh hydrogels. c) Stress–strain curves of the complex hydrogels after being placed for 72 h under the relative humidity of 32–88%. d) Stress–strain curves of the film with an m_PAM_/*m*_calcium chloride_ mass ratio of 5:10 after being placed for *x* (*x* = 1–24) h in a vacuum environment at −60 °C. e) Practical stress–strain curves of the film with an *m*_PAM_/*m*_calcium chloride_ mass ratio of 5:10 at room temperature during the 65% relative humidity. f) Stress–loading/unloading curves of the complex hydrogel with an *m*_PAM_/*m*_calcium chloride_ mass ratio of 5:10 after two weeks of standing under the relative humidity of 65%. g) The stress–strain curves of the fresh hydrogels with an *m*_PAM_/*m*_calcium chloride_ mass ratio of 5:10 healed at 50 °C for 6, 12, and 24 h. The measuring conditions for the tensile test were a diameter of 5 mm, gauge length of 5–20 mm, and loading rate of 50 mm min^−1^.

As shown in **Figure** **2**a, a maximum fracture test was carried out for the self‐prepared hydrogels of 2 mm thickness, 4 mm width, and 5–20 mm gauge length at a loading rate of 100 mm min^−1^. The fresh HAH@CTAB@CaCl_2_ sample with a water content of 50 ± 5 wt% can be stretched to a much higher strain before fracturing than can typical HAH hydrogel. A maximum fracture strain up to 4700 ± 10% can be achieved for the gel with an *m*_PAM_/*m*_calcium chloride_ mass ratio of 5:2 and the soft material can self‐recover to its original length within one hour through stress release. Incredibly, the transparent hydrogel with an *m*_PAM_/*m*_calcium chloride_ mass ratio of 5:10 can even be stretched to over 85 times its original length without breaking. In comparison, the typical hydrophobic associating hydrogels with similar dimensions exhibit a maximum elongation of only 1000–3500%.^[^ ^17^ ^]^ We propose that the high stretchability of the HAH@CTAB@CaCl_2_ material can be attributed to the dynamically balanced Ca ← O═C─R coordination bond, concluding intrachain and interchain metal–ligand interactions. Intrachain metal–ligand interactions cause PAM chains to fold, allowing larger chain extensions to occur during breaks, while interchain metal–ligand interaction leads to 3D‐crosslinking and repeated bonding/breakage between main chains through chain sliding. The dynamic nature of the soft material allows Ca ← CONH_2_ coordination bond to break and re‐form during stretching process, which leads to the unfolding and sliding of the PAM polymer chains and gives the amazing stretchability of the hydrogel.

Mechanical performances of hydrogels are closely related to water content.^[^ ^18^ ^]^ The water content of all homogeneous HAH@CTAB@*x*CaCl_2_ hydrogels with different mass ratios of *m*_PAM_/*m*_calcium chloride_ (5:2, 5:4, 5:6, 5:8, and 5:10) is adjusted to 25 ± 5% by hierarchical heating, and tensile stress curves of the films are shown in Figure S1, Supporting Information. The maximum fracture of the hydrogels are equal to 1300%, 1820%, 2100%, 2600%, and 3050%, respectively, with the fracture energy of 210, 181.9, 144, 155, and 87 kJ m^−2^. To test the correlation between mechanical properties and water content, the super‐elastic hydrogels with an *m*_PAM_/*m*_calcium chloride_ mass ratio of 5:10 were placed at 25 °C for 72 h under the relative humidity of 32–88%. As shown in Figure 2c, the water content of the hydrogels increases from 45.9% at a 32% relative humidity to 57.2% at a relative humidity of 88%. The ultimate tensile length increases significantly with the increase of water content, but the breaking strength decreases rapidly. For example, the tensile length of the sample placed under a 88% relative humidity can reach 12 500%, which only corresponds to a tensile stress of 7.3 kPa. The hydrogels with an *m*_PAM_/*m*_calcium chloride_ mass ratio of 5:10 were freeze‐dried in a vacuum environment at −60 °C to further examine the relationship between mechanical properties and water content, as shown in Figure S2, Supporting Information and Figure 2d. The water content remains at ≈44% even if the hydrogel is freeze‐dried for 24 h, and the corresponding breaking strength and elongation are 790 kPa and 2060%, respectively. Therefore, coordination bond provides multiple energy dissipation mechanisms of breaking, exchanging and re‐formtion, which makes the hydrogel more elastic. The transmittance of hydrogel is positively correlated with water content. For all fresh HAH@CTAB@*x*CaCl_2_ (*x* = 0–10) materials with the thickness of 2 mm, a high transmittance value of more than 85% is obtained at a wavelength of 550 nm. After being placed at room temperature for two weeks during a 65% relative humidity, the transmittance drops to 0.3–49.5% in Figure S3, Supporting Information. Compared with hydrogen bonds, coordination bonds with a stronger water‐locking effect support the 3D network of the hydrogels, thereby obtaining high light transmittance.

Complex hydrogels were placed at room temperature during a relative humidity of 65% to investigate the stability or reliability of mechanical properties. After being placed for three days, the hydrogel material with an *m*_PAM_/*m*_calcium chloride_ mass ratio of 5:4 reaches a dynamic equilibrium of vapor adsorption‐desorption in Figure S4, Supporting Information, and the corresponding fracture length and strength of the gel are about respectively 2000% and 800 kPa, respectively. When we increase the amount of CaCl_2_ to 10 g, it takes more than 7 days for the as‐prepared hydrogel to reach the adsorption‐desorption equilibrium in the same environment, and the corresponding limit stretch length exceeds 2700%. In contrast, PAM chains of the typical HAH gel undergo intramolecular and intermolecular physical cross‐linking, because hydrogen bonds are difficult to continuously lock water molecules even at room temperature. It only takes 24 h to change from a transparent projectile to a milky white hard block. The self‐recovery and fatigue resistance of the hydrogel are also investigated by tensile cycle test. The continuous 10 times tensile cycling curves without any residence time are shown in Figure 2f for the complex hydrogel with an *m*_PAM_/*m*_calcium chloride_ mass ratio of 5:10. Almost coincident stretching curves indicate that the complex hydrogel has good self‐recovery and anti‐fatigue damage, while the Figure 2f and embedded graph reveal the excellent self‐healing ability of the fresh interface of the hydrogel.

#### 2.2.2 Coordination Chemical Bond

**Figure 3:**
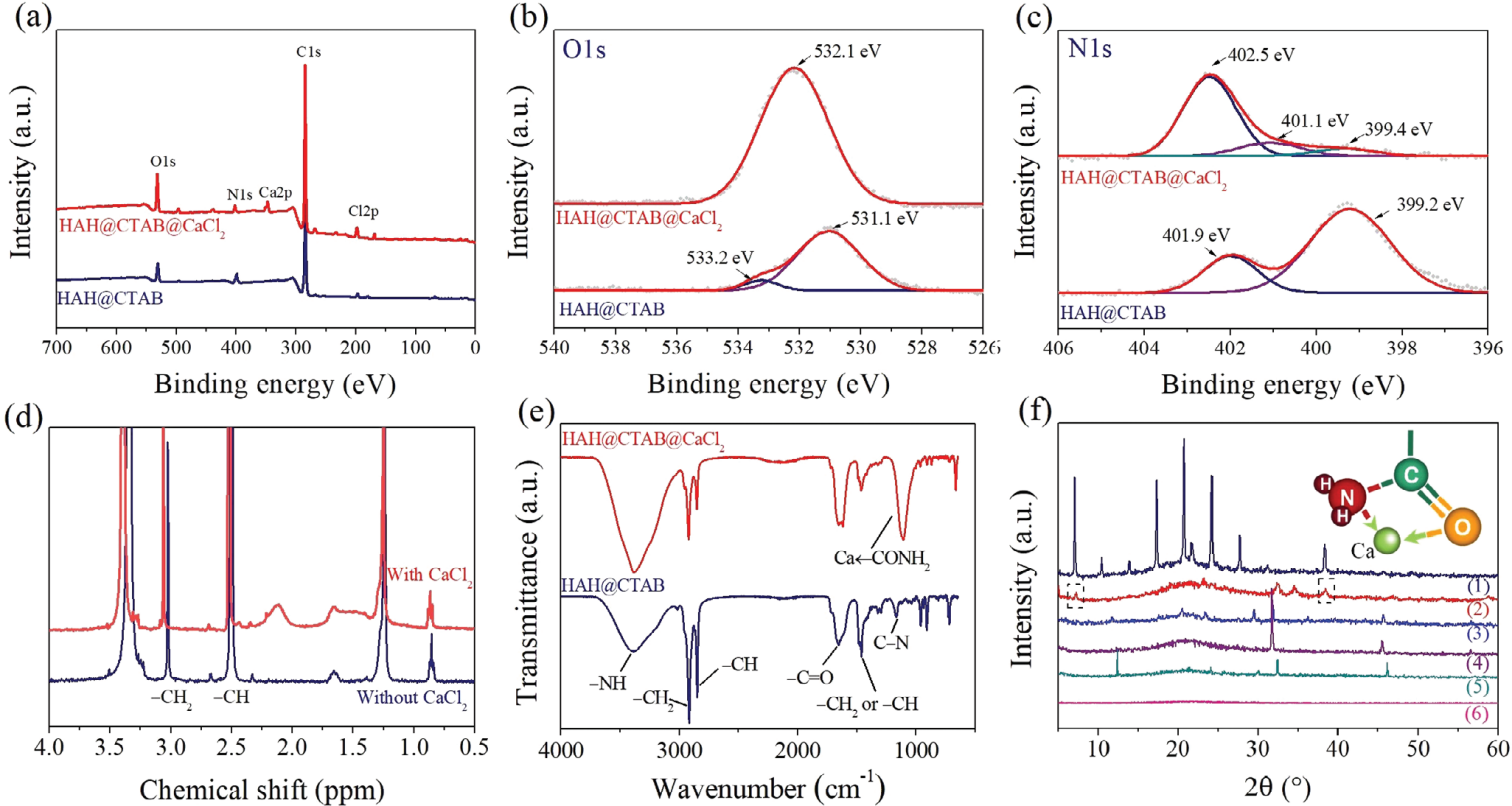
Evidence of coordination chemical bond between calcium metal ion and amide ligand. a) The survey XPS spectra of the hydrogels with/without Ca‐ion. b) High‐resolution XPS sepctra of O 1s. c) High‐resolution XPS sepctra of N 1s of the as‐prepared hydrogels. d) ^1^H CP‐MAS NMR spectra. e) FT‐IR spectra of the anhydrous HAH@CTAB and HAH@CTAB@CaCl_2_. f) XRD spectra of the anhydrous materials with CaCl_2_ mass of 0 g (1), 2 g (2), 4 g (3), 6 g (4), 8 g (5), and 10 g (6).

In order to understand the intrinsic principle of improving mechanical properties of HAH@CTAB@CaCl_2_ gel, X‐ray photoelectron spectroscopy (XPS), ^1^H CP/MAS NMR, Fourier‐transform infrared spectroscopy (FT‐IR), and X‐ray diffraction (XRD) pattern were carried out for the as‐prepared HAH@CTAB@CaCl_2_ gel in **Figure** **3**. As‐prepared HAH gels with/without CaCl_2_ exhibit three intense peaks at 285, 400, and 531 eV corresponding to C 1s, N 1s, and O 1s core levels in Figure 3a, respectively. The Ca 2p core spectrum of the HAH@CTAB@CaCl_2_ gel can be cure‐fitted to two peaks at 351.6 and 348.0 eV that belong to the inorganic calcium salt^[^ ^19^ ^]^ and coordination bond between calcium ion and amide group, respectively (Figure S5, Supporting Information). Compared with that of the survey spectrum of the HAH@CTAB, the intensity of the O 1s peak of the gel with CaCl_2_ is significantly increased in Figure 3b, and the peak shifts to 532.1 eV, which belong to the Ca← O═C─ bond. As shown in Figure 3c, the N 1s spectrum of the HAH@CTAB@CaCl_2_ gel can be fitted to three peaks, centered at the binding energy of 402.5, 401.1, and 399.4 eV, attributing to N─C,^[^ ^20^ ^]^ Ca ← NH_2_─C─ and N─H groups,^[^ ^21^ ^]^ respectively. Therefore, both the carbonyl (─C═O) and amino (─NH_2_) groups of the PAM can coordinate with calcium ions (II).

Formation of the Ca ← CONH_2_ coordination bond is confirmed by a ^1^H‐NMR hydrogen spectrometer, as shown in Figure 3d. For the typical HAH gel with an *m*_PAM_/*m*_calcium chloride_ mass ratio of 5:0, the signals at 3.02 and 2.50 ppm are assigned to ─CH_2_ and ─CH of the PAM chain,^[^ ^22^ ^]^ respectively. The Ca ← CONH_2_ coordination bond is evidenced by the new adsorption peak at 2.12 ppm, and the signal peaks of the ─CH_2_ and ─CH are shifted a little to the left. DSC results (Figure S6, Supporting Information) further reveal the coordination information of calcium ion and amide group. Acrylamide (AAM) has a very obvious endothermic peak at 80.9 °C and the enthalpy value is as high as 598 J g^−1^, which corresponds to the sublimation process of AAM. However, it is impossible to observe any endothermic peaks in the AAM@CaCl_2_ mixture within the same temperature range.

FT‐IR spectroscopy is one of the most popular methods for detecting chemical bonds or functional groups of organic materials. As shown in Figure 3e and Figure S7, Supporting Information, the FT‐IR spectra of both anhydrous and moist HAH@CTAB@CaCl_2_ gels presents a new characteristic absorption peak at 1110 cm^−1^, which is attributed to Ca ← CONH_2_. It is impossible to observe obvious diffraction peaks for all fresh HAH@CTAB@*x*CaCl_2_ (*x* = 2, 4, 6, 8, and 10) hydrogels with a water content of 50 ± 5 wt% (Figure S8, Supporting Information), because the coordination bond of calcium ion‐amide group and the hydrogen bond of water‐amide limit the intramolecular/intermolecular bonding of the PAM main chain.^[^ ^23^ ^]^ The gel was freeze‐dried in a vacuum environment at −60 °C for 48 h, and the corresponding XRD test results were shown in Figure 3f. The milky white HAH gel (1) shows a series of diffraction peaks, indicating that PAM polymer crystallizes through intramolecular and/or intermolecular interaction forces. The weak crystallization peaks of PAM chains are observed at 7.3° and 38.4° for the hydrogel with a mass ratio of PAM to CaCl_2_ of 5:2. However, the characteristic diffraction peaks disappear for the anhydrous projectiles with the calcium chloride mass of 4–10 g due to the coordination bond maintaining the 3D network structure of the gel.

### 2.3 Anti‐Freezing and Heat Resistance of the As‐Prepared Hydrogel

**Figure 4:**
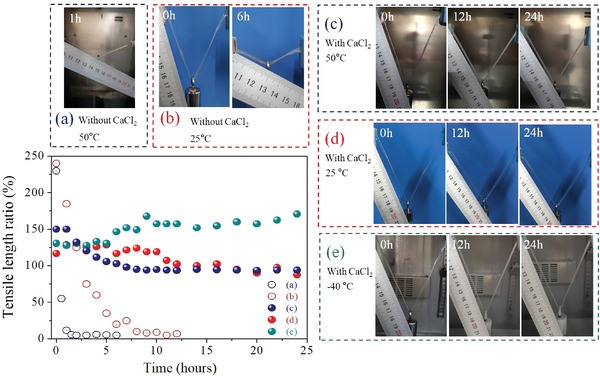
Anti‐freezing and anti‐heating performances of the HAH@CTAB@CaCl_2_ gel with a diameter of 5 mm and an effective length of 60–80 mm under an external force of 0.2–0.5 N in a wide temperature window of −40–50 °C. Note: As‐prepared hydrogels are not subjected to external force during the static process, which is to prevent unnecessary deviation of the test results due to water loss. Figures (a–e) are actual photos of the hydrogels being stretched at different temperatures.

**Figure 5:**
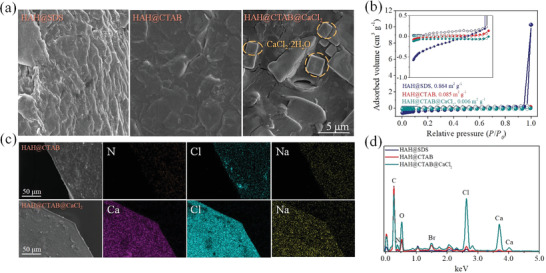
Characterization of the HAH@SDS, HAH@CTAB, and HAH@CTAB@CaCl_2_ hydrogels. a) FE‐SEM images of the anhydrous gels. b) BET surface area distribution of the HAH gels. c) Elemental mapping by EDX for the hydrogels revealing a uniform distribution of Ca and Cl. d) EDS curves of the as‐prepared materials.

Tensile resistance test was carried out in situ for the complex hydrogel with a diameter of 5 mm and an effective length of 60–80 mm in a temperature window of −40–50 °C, and corresponding test results were displayed in **Figure** **4**. The initial stretched length of the typical hydrogel with an *m*_PAM_/*m*_calcium chloride_ mass ratio of 5:0 is 240% under an external force of 0.5 N, but the stretch ratio drops to only 10% after 8 h of storage even at room temperature. The tensile rate of the linear HAH@CTAB hydrogel under the same load decreases from 230% to 11.8% after being placed at 50 °C for only 1 h. **Figure** **5** shows the microscopic morphology and N_2_ adsorption‐desorption curve of the as‐prepared hydrogels. Anhydrous HAH gel is impossible to observe micro‐submicron pores, and the calculated Brunauer–Emmett–Teller (BET) surface area is 0.006–0.864 m^2^ g^−1^, which is farther lower than the typical porous gels reported in literature (≥50 m^2^ g^−1^).^[^ ^24^ ^]^ It can be seen from Figure S9, Supporting Information that the weight loss rate of the typical HAH@CTAB material is as high as 31 mg per g‐HAH for 1 h, accompanied by a large surface area shrinkage of 44%. As a result, hydrogen bond as a typical weak interaction force cannot lock water molecules during the low relative humidity of 65%, causing the super‐elastic properties of the HAH gel to fail.

**Figure 6:**
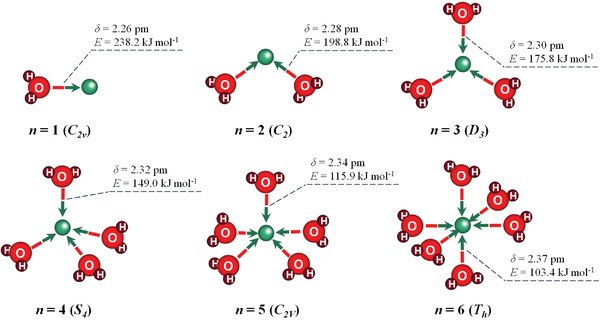
Lowest energy structure of the [Ca(H_2_O)*_x_*]^2+^ (*x* = 1–6) clusters. Note: The red, dark red, and dark green balls represent oxygen, hydrogen, and calcium, respectively.

The super‐elastic hydrogel with an *m*_PAM_/*m*_calcium chloride_ mass ratio of 5:10 maintains stable mechanical properties, including strength, elongation, and recoverability, in a wide temperature range of −40–50 °C. Stretch length increases slowly from 130% to 157% at −40 °C for 12 h, with the mass increase rate of 5.3 mg per g‐gel for one hour (Figure S10, Supporting Information). The large temperature difference (≈65 °C) between the internal and external environments promotes the formation of frost/dew on the surface of the hydrogel, resulting in slight changes in the parameters of the modified hydrogel. As far as we know, calcium metal ions^[^ ^25^ ^]^ can bond with water molecules through coordination bonds (Ca ← OH) to form hydrated ion clusters [Ca(H_2_O)*_x_*]^2+^ (*x* = 1–6). Compared with the low bonding energy (21 kJ mol^−1^) of hydrogen bonds,^[^^]^ that of the calcium hydrate ions is as high as 103.4 ∼ 238.2 kJ mol^−1^ in **Figure** **6**, which significantly reduces the crystallization temperature of liquid water. For example, the freezing point of the typical 29.9 wt% calcium chloride solution drops from 0 to −55 °C.^[^ ^26^ ^]^ The super‐elastic hydrogels were placed at different relative humidity for 3 days, and the corresponding DSC curves were shown in Figure S11, Supporting Information. The higher the water content of the gel, the higher the freezing point. Even so, the freezing point of the hydrogel after fully absorbing moisture under the 88% relative humidity is lower than −55 °C, while the crystallization peak temperature is only −82 °C at a 72% relative humidity. Based on the strong interaction among water molecules, calcium ions, and gel network, water molecules are not easy to form nucleation, which results in the retention of unfrozen water at very low temperatures.^[^ ^27^ ^]^ For example, super‐cooling can be observed down to −35–−38 °C for ultra‐pure water by avoiding nuclei as well as external perturbations.^[^ ^28^ ^]^ According to the Figure 2d, water content of the HAH@CTAB@CaCl_2_ with an *m*_PAM_/*m*_calcium chloride_ mass ratio of 5:10 is still as high as 44% after be drying at −60 °C for 24 h in a vacuum environment. In contrast, the hydrogel without calcium chloride has a water content of less than 4% under the same treatment conditions in Figure S12, Supporting Information. Based on the anti‐freeze and water retention mechanism of calcium ions, the self‐prepared hydrogel can continuously maintain excellent mechanical properties at −40 °C.

The HAH@CTAB@CaCl_2_ gel can also resist the damage caused by a high temperature environment of 50 °C, based on the constant strain value of 95% after 8 h, which is basically consistent with the mass/volume change law of the modified hydrogel in Figures S13,S14, Supporting Information. We placed the complex hydrogel under the extreme condition of 100 °C for 12 h to investigate its water retention performance. The same amount of pure water is completely evaporated within only one hour, but the water content of the high‐quality hydrogel with thickness of about 2 mm is controlled within 15–25% of total gel mass for 12 h. The complex hydrogel that has withstood the test at 100 °C still exhibits a maximum fracture length of more than 23 times in Figure S15, Supporting Information. Weight change of the saturated calcium chloride solution at 90 °C for 8 h is only limited to 3.3% in Figure S16, Supporting Information, while the surface of the aqueous solution is covered with a transparent and dense crystal film (CaCl_2_·2H_2_O or CaCl_2_·4H_2_O).^[^ ^29^ ^]^ As shown in Figure 5a, a cubic shape of CaCl_2_·2H_2_O with a side length of 1–2 µm can be observed on the surface of the thoroughly dried gel. In other words, [Ca(H_2_O)*_x_*]^2+^ hydrated ion clusters restrain the freely moving water molecules, resulting in a significant reduction in the critical relative humidity of the aqueous solution; while the crystalline hydrate acts as a barrier to further slow down the loss of water molecules. The dual water retention mechanisms provided by calcium metal ions support the gel to maintain good mechanical performances under an extreme high temperature condition.

### 2.4 Flame Retardancy of the As‐Prepared Hydrogel

**Figure 7:**
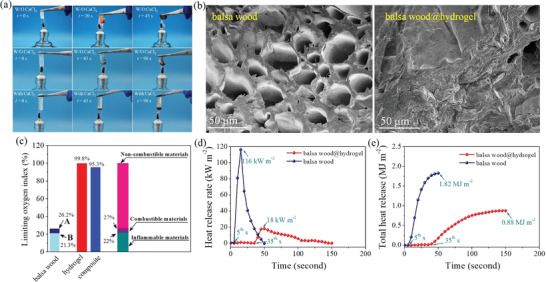
Anti‐combustion performance test of the hydrogels. a) Digital photos lit under an open flame with the temperature of 400–500 °C. b–e) FE‐SEM images (b), LOI value (c), heat release rate (d), and total heat release (e) of the porous balsa wood and as‐prepared hydrogel. Note: The LOI value of balsa wood (A) in the natural state is 26.2%. When balsa wood (B) is placed at 25 °C for 7 days during the 50% relative humidity, the LOI value drops to 21.3%.

Flame retardancy of the self‐prepared hydrogels with/without CaCl_2_ was evaluated in **Figure 7** by the actual heating combustion, thermogravimetric (TG) analysis, LOI, and cone calorimetry test results. Anhydrous HAH@CTAB gel, freeze‐dried in a vacuum environment at −60 °C for 48 h, is completely burned in only 45 s in Figure 7a, and the TG curve further confirms the flammability of the dry gel in Figure S17, Supporting Information. Two‐thirds of the wet hydrogel with an *m*_PAM_/*m*_calcium chloride_ mass ratio of 5:0 is burned within 90 s, but there is no open flame during the entire burning process based on a large evaporation enthalpy of water (40.63 kJ mol^−1^ at 100 °C).^[^ ^30^ ^]^ The soft material with an *m*_PAM_/*m*_calcium chloride_ mass ratio of 5:10 still exhibits an excellent flame retardancy even after being kept for two weeks during a relative humidity of 65%. Only 5 mm length is burned for the complex hydrogel in 90 s, accounting for about one‐tenth of the total length. As shown in Video S1, Supporting Information, the complex hydrogel with thickness and length of 1 and 140 mm, respectively can withstand a continuous open flame test for 15 min. We encapsulated the hydrogel in porous balsa wood material to further test the flame retardant properties. LOI values of the HAH@CTAB@CaCl_2_ and its composite are equal to 99.8% and 95.3% respectively, which is significantly higher than 21.3% of balsa wood. As shown in Figure 7d, the heat release rate curve of porous wood presents a sharp peak from 5th to 50th s, with a maximum heat release power of 116 kW m^−2^ for 1 g material. For the balsa wood@hydrogel material, ignition time is delayed to 35th s, and corresponding limit heat release power and total heat release are only 18 kW m^−2^ and 0.88 MJ m^−2^, respectively. Dual flame‐retardant mechanisms, concluding the endothermic effect of liquid water evaporation and the barrier effect of calcium chloride hydrate on oxygen, make the complex hydrogel a veritable non‐flammable material.

## 3 Conclusion

In this work, we developed a straightforward design strategy to fabricate a novel type of fully physically linked hydrophobic associating hydrogel, consisting of the hydrogen bond crosslinked PAM‐water and coordination bond crosslinked PAM‐Ca (II). The dynamic balance of the metal–ligand bond allows it to be rapidly broken and re‐formed during stretching, which leads to unfolding and sliding of the PAM chains. The as‐prepared hydrogel can be stretched to 136 times its original length, which is significantly better than the value reported in literature.^[^ ^17^ ^]^ The strong coordination bonds of PAM chain‐calcium (II) and water‐calcium (II) firmly fix the water in the polymer network, so that the high‐quality hydrogel presents excellent overall performances of anti‐freezing, heat resistance, flame retardancy, and long‐lasting mechanical stability. It is believed that the complex hydrogel may be an ideal candidate for designing biological muscles and medical drug carriers, repairable building materials, and vulcanized rubber substitutes.

## 4 Experimental Section

### Materials

All experimental chemicals and solvents were of analytical grade and used without further purification. AAM (99.0%), sodium dodecyl sulfate (SDS, 97%), hexadecyl trimethyl ammonium bromide (CTAB, 99.0%), hexadecyl methacrylate (HMA, 95%), sodium chloride (NaCl, 99.5%), calcium chloride (CaCl_2_, 96%), potassium persulfate (KPS, 99.5%) and *N,N,N,N*‐tetramethylethylenediamine (TEMED, 99.5%) were supplied by Aladdin (Shanghai, China).

### Preparation Principle of HAH Gels

Super‐elastic HAH was synthesized by in situ polymerization.^[^ ^31^ ^]^ 1 g CTAB as a surfactant, *x* g CaCl_2_ (*x* = 0–10), and 0.406 g NaCl as a co‐solvent for HMA hydrophobic monomer were dissolved in 20 mL deionized water, and stirred until the solution was clarified. 0.254 mL HMA was added to the prepared micellar solution. After the solution was fully stirred at room temperature for 5 h, 5 g AAM and 0.02 g KPS chain initiator were added to the highly dispersed micellar solution. After stirring for 1 h, 0.052 mL TEMED as a crosslinking agent for AAM was added. Finally, the well‐stirred mixture was poured into the customized glass mold and aged in a vacuum drying oven at 50 °C for 12 h to obtain transparent HAHW gel with 45 ± 5 wt% of liquid water. According to the composition, the as‐prepared hydrogels were referred to as HAH@SDS, HAH@CTAB, and HAH@CTAB@CaCl_2_.

### Preparation of the Balsa Wood@Hydrogel Composite

The long strips of balsa wood (100 mm × 30 mm × 3 mm) were placed in an Erlenmeyer flask and vented in a vacuum environment at 25 °C for 6 h. Then, the fresh sol with an *m*_PAM_/*m*_calcium chloride_ mass ratio of 5:10 was poured into the Erlenmeyer flask through the separatory funnel and kept stirring for 24 h. In the next step, the sol was aged at 50 °C for 12 h to obtain the balsa wood@hydrogel composite sample. Finally, the gel coated on the surface of the balsa wood was removed after the composite was placed at 25 °C for 14 days during a relative humidity of 65%. The loading capacity (*β_1_*) of the hydrogel is calculated by the following formula:

(1) $$\beta_{1}=\left(M_{\mathrm{cm},1}-M_{\mathrm{bw}}\right)/M_{\mathrm{bw}}$$

where *M*_cm,1_ and *M*_bw_ are the mass of composite material and balsa wood, respectively.

Relative content (*β*_2_) of anhydrous gel is determined by the equation below:

(2) $$\beta_{2}=\left(M_{\mathrm{cm},2}-M_{\mathrm{bw}}\right)/M_{\mathrm{bw}}$$

where *M*_cm,2_ is the weight of the anhydrous composite. According to the test, *β_1_* and *β_2_* were equal to 55 ± 5% and 25 ± 5% respectively.

### Material Characterization

FT‐IR spectra were recorded on a FT‐IR (Nicolet 5700, USA) using freeze‐dried hydrogel pellet (4000–500 cm^−1^). The surface morphology investigation was carried out on a field emission scanning electron microscope (FE‐SEM, Hitachi S4800) and energy‐dispersive spectroscopy mapping (EDS, Bruker Nano XFlash Detector 5030). Before the FE‐SEM investigation, the samples were sputtered with gold. A Kratos AXIS‐SUPRA spectrometer was used to analyze the XPS (Zetasizer Nano S90) of the materials. Powder XRD measurements were performed using Cu K*α* (*λ* = 0.154056 nm) radiation with a scan rate of 4° min^−1^ and a step size of 0.03° in the 2*θ* range of 5° to 60°. The ^1^H CP/MAS NMR spectra were measured on a JEOL JNM‐ECZ‐600R spectrometer. The specific surface area of the composite material was measured through nitrogen adsorption at 77 K using a Micromeritics ASAP 2040 Analyzer in the range of the relative pressures *P*/*P*_0_ = 0.06–0.99 with a step of 0.015. Q500 equipment was used for TG analysis with a heating rate of 20 °C min^−1^ from 35 to 800 °C in the air. The transmittance of the hydrogel was measured with a commercial tester (LS116, China) at a wavelength of 550 nm.

### Flame Retardancy Test

A cone calorimeter was used to test the flame retardancy of the balsa wood@hydrogel composite (100 mm × 30 mm × 3 mm) according to the ISO 5660‐1. All sides of the sample except the heating surface was wrapped with aluminum foil and placed it horizontally on the stainless steel sample holder, and the heat flux was equal to 50 kW m^−2^. Based on ISO 4589‐1, the LOI was measured for the composite at 23.1 °C under a 50% relative humidity.

### Mechanical Tests

Tearing testing was carried out with a commercial test tensile tester (MTS E44) with a 100 N load cell. The hydrogels were cut into standard dumbbell shapes with 2 mm thickness, 4 mm width, and 5–20 mm gauge length. The two ends of the materials were clamped, in which the one end was fixed, while the other one was pulled at 100 mm min^−1^. The tearing energy (*E*) is defined as the work required to tear a unit area, as estimated by^[^ ^6^ ^]^

(3) E=2F/λ

where *F* is the average force of peak values during steady‐state tear, and *λ* is the width of the specimen.

## Conflict of Interest

The authors declare no conflict of interest.

## Supporting information

Supporting Information

Supporting Information

## Data Availability

Research data are not shared.
