# Supplementary material for: A Smart Design Strategy for Super‐Elastic Hydrogel with Long‐Term Moisture, Extreme Temperature Resistance, and Non‐Flammability
Source: Adv Sci (Weinh). 2021 Jun 19;8(16):2100320. doi: 10.1002/advs.202100320 (PMC8373105; doi:10.1002/advs.202100320)
Supplement: Supplementary file 1 — Supporting Information [file ADVS-8-2100320-s001.pdf]

WILEY-VCH

© 2021 Wiley-VCH GmbH

ADVANCED SCIENCE
Open Access

Supporting Information

for *Adv. Sci.*, DOI: 10.1002/advs.202100320

A Smart Design Strategy for Super-Elastic Hydrogel with Long-Term Moisture, Extreme Temperature Resistance and Non-Flammability

Haiquan Zhang†, Zijing Liu†, JunPing Mai, Ning Wang, Houji Liu, Jie Zhong and Xianmin Mai**

© 2021 Wiley-VCH GmbH

Supporting Information

A Smart Design Strategy for Super-Elastic Hydrogel with Long-Term Moisture, Extreme Temperature Resistance and Non-Flammability

Haiquan Zhang†, Zijing Liu†, JunPing Mai, Ning Wang, Houji Liu, Jie Zhong and Xianmin Mai**

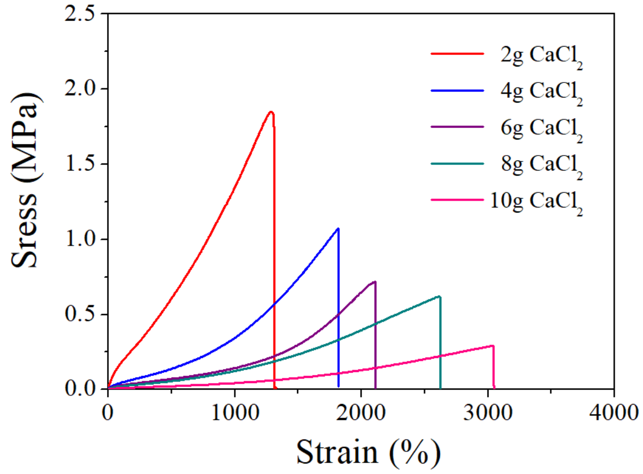

Figure S1. Stress-strain curves of the complex hydrogel with water content of 25 ± 5%.

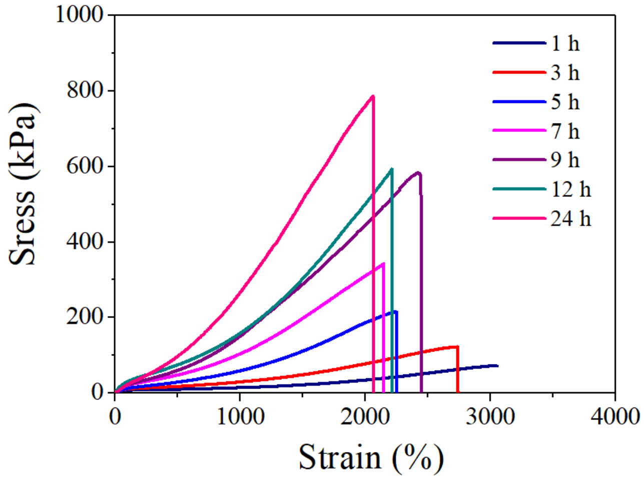

Figure S2. Stress-strain curves of the film with a $m_{PAM}/m_{calcium\ chloride}$ mass ratio of 5:10 after standing for *x* (x =1 – 24) hours in a vacuum environment at -60 °C.

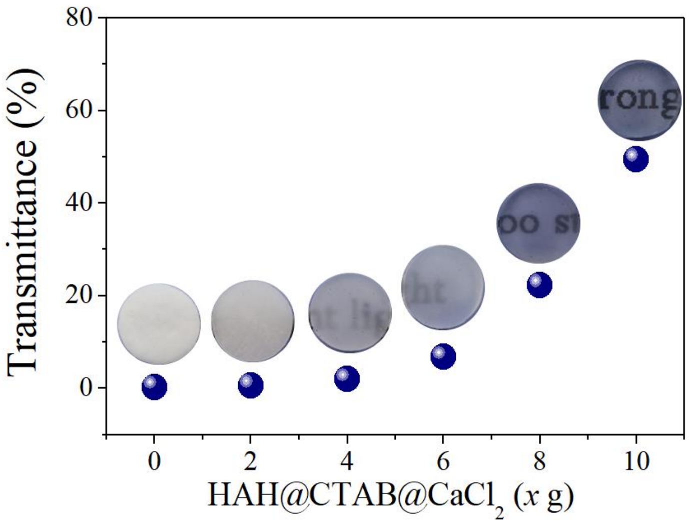

Figure S3. The relationship curve between transmittance and calcium chloride mass for the HAH samples with the thickness of 2 mm, when the hydrogels are placed at room temperature for 14 days during the relative humidity of 65%.

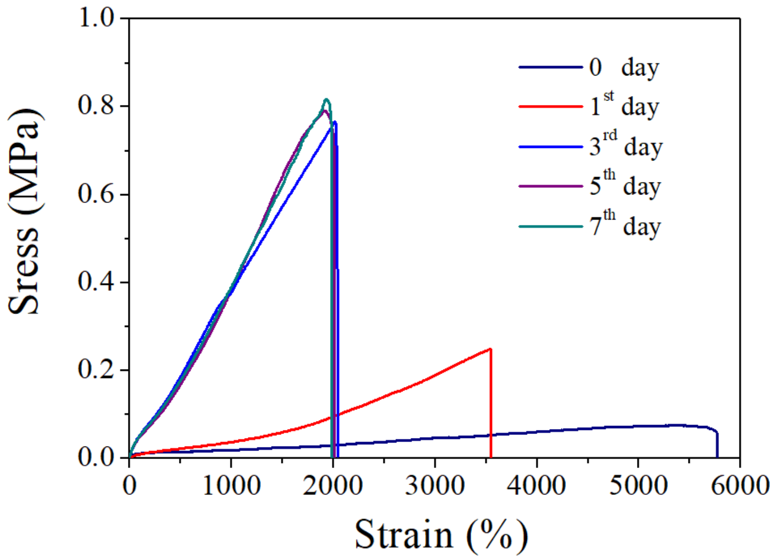

Figure S4. Practical stress-strain curves of the film with a $m_{PAM}/m_{calcium\ chloride}$ mass ratio of 5:4 at room temperature during the 65% relative humidity.

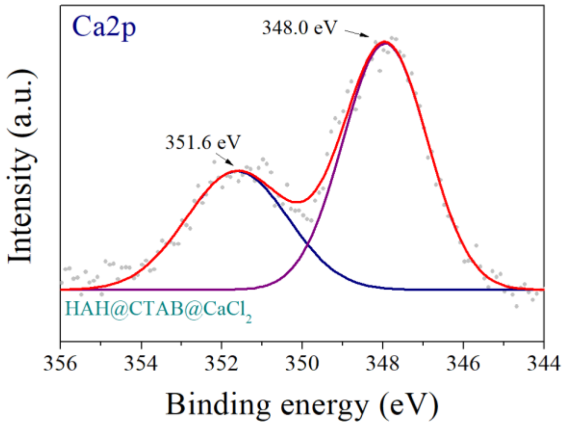

Figure S5. High-resolution XPS spectrum of the Ca-ion of the as-prepared hydrogel.

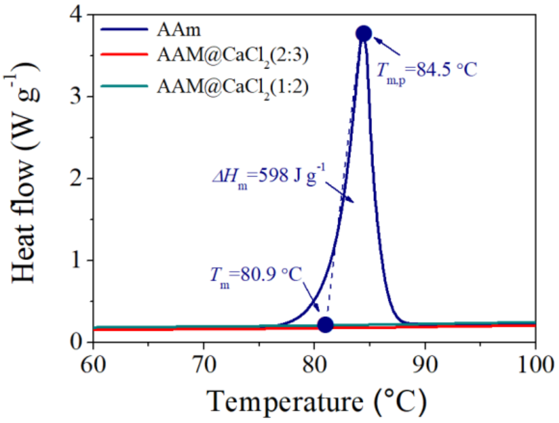

Figure S6. DSC curves of the AAM, $AAM@CaCl_2$ (2: 3) and $AAM@CaCl_2$ (1: 2) materials.

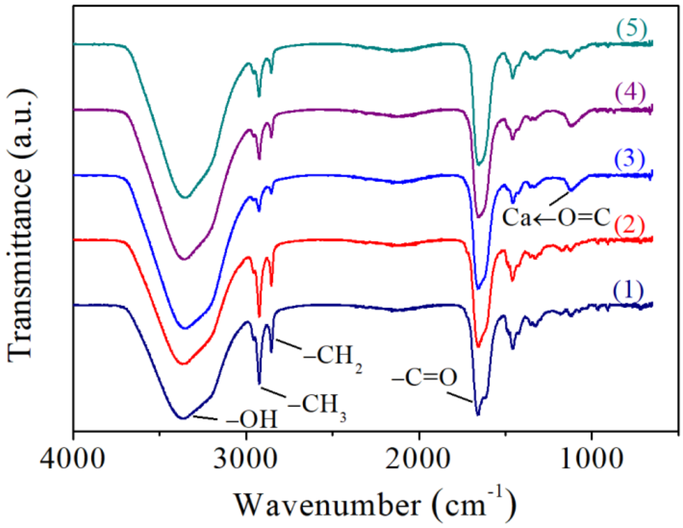

Figure S7. FT-IR profiles of the wet HAH@CTAB@$x$$CaCl_2$ (x = 2 – 10) materials. (1) 2 g $CaCl_2$; (2) 4 g $CaCl_2$; (3) 6 g $CaCl_2$; (4) 8 g $CaCl_2$; and (5) 10 g $CaCl_2$.

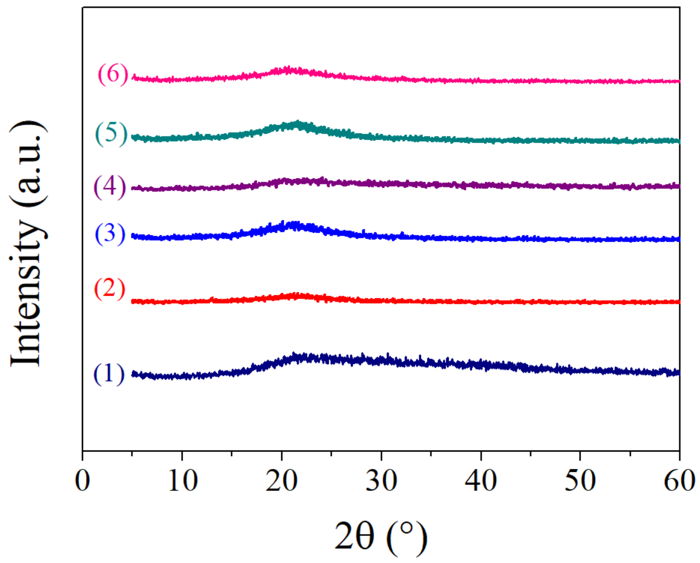

Figure S8. XRD spectra of the wet HAH@CTAB@xCaCl$_2$ (x = 0 – 10) materials. (1) 0 g CaCl$_2$; (2) 2 g CaCl$_2$; (3) 4 g CaCl$_2$; (4) 6 g CaCl$_2$; (5) 8 g CaCl$_2$; (6) 10 g CaCl$_2$.

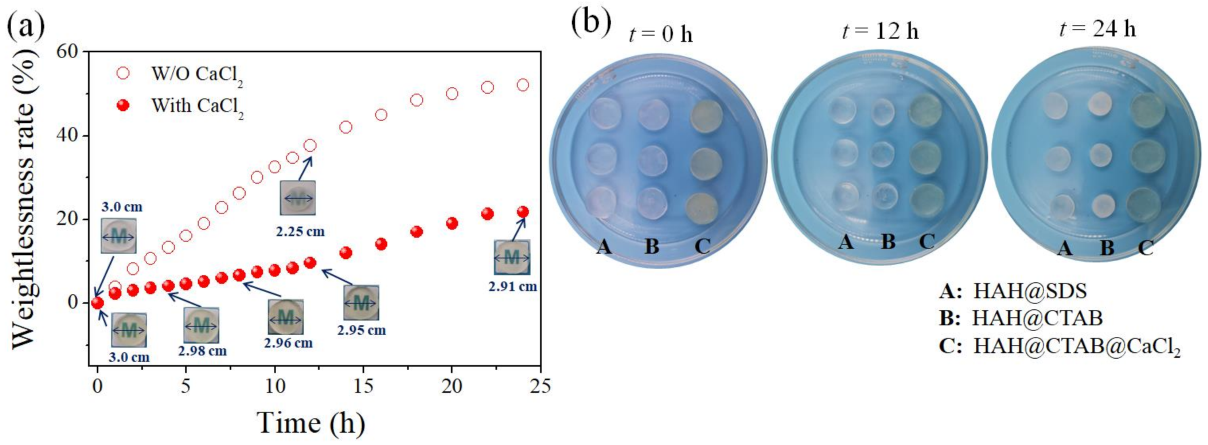

Figure S9. Adsorption-desorption curves of the hydrogels with/without CaCl$_2$ at room temperature at a relative humidity of 65%.

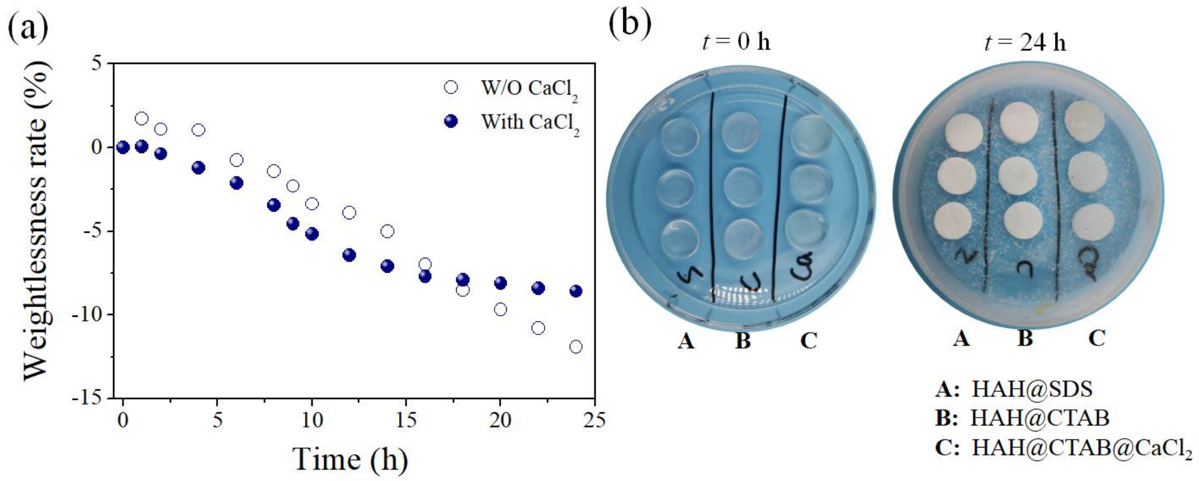

Figure S10. Adsorption-desorption curves of the super-elastic hydrogels at low temperature of -40 °C for 24 hours.

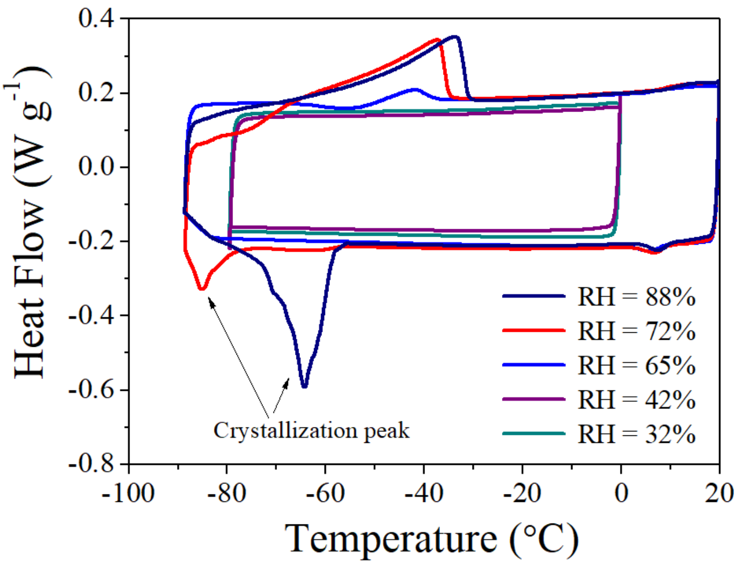

Figure S11. DSC curves of the complex hydrogels after standing for 72 hours under the relative humidity of 32% – 88%.

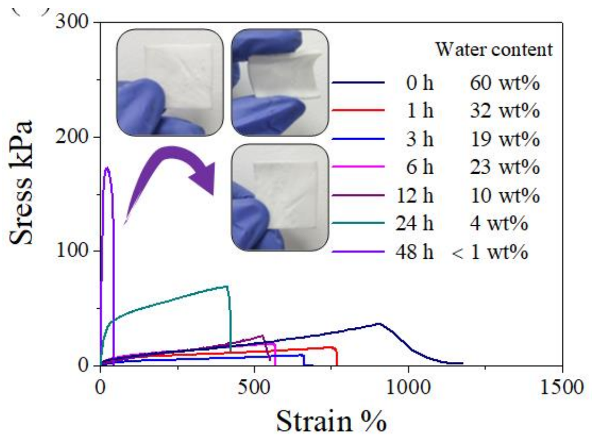

Figure S12. Mechanical properties and water content of calcium chloride-free hydrogel after being drying at -60 °C in a vacuum environment.

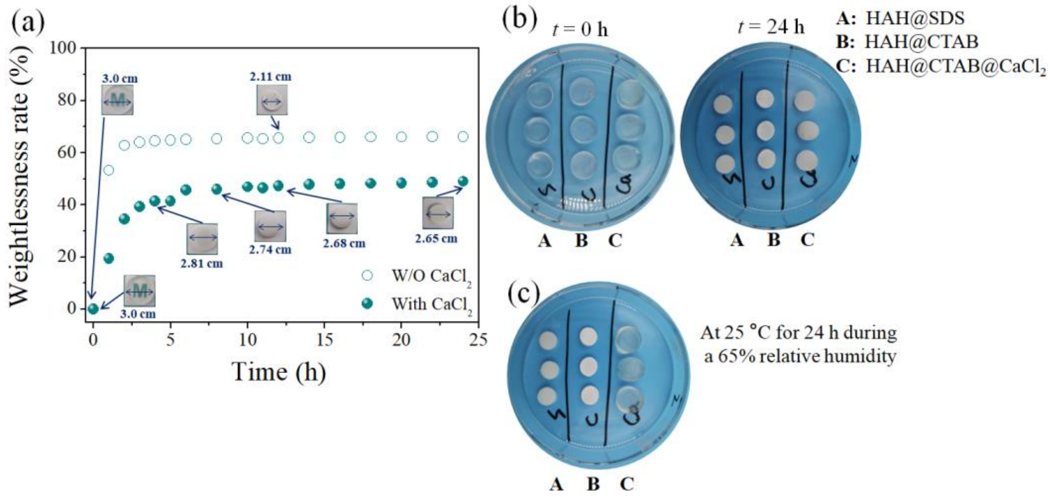

Figure S13. Adsorption-desorption curves of the super-elastic hydrogels at 100 °C for 24 hours.

According to Figure S13, the volume shrinkage of the HAH@CTAB@$CaCl_2$ gel is significantly lower than that of the calcium chloride-free sample at 100 °C for 24 hours. In addition, the anhydrous HAH@CTAB@$CaCl_2$ sample is placed at room temperature for 24 hours under a 65% relative humidity, and it became a transparent super-elastic material again. In contrast, the sample without calcium chloride is still a hard white block.

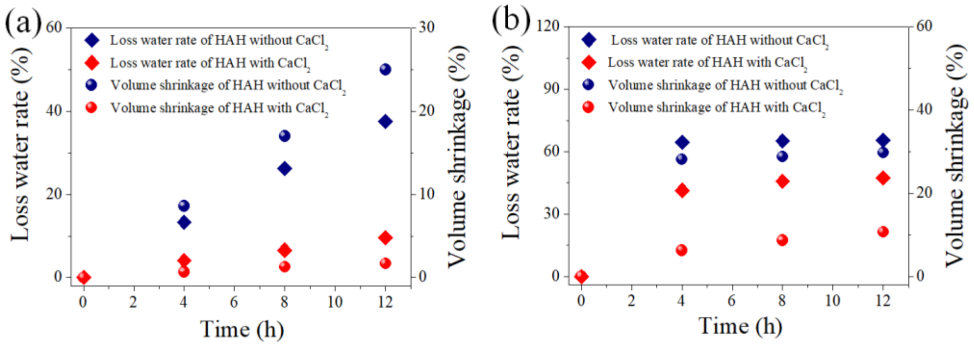

Figure S14. Relationship between water loss rate and volume shrinkage at 25 (a) and 100 (b) °C.

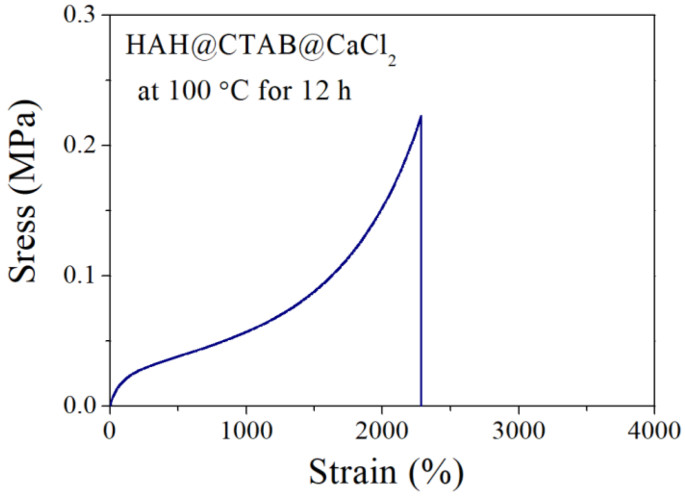

Figure S15. Tensile stress curves of the HAH@CTAB@$CaCl_2$ material after 12 hours of heating at 100 °C.

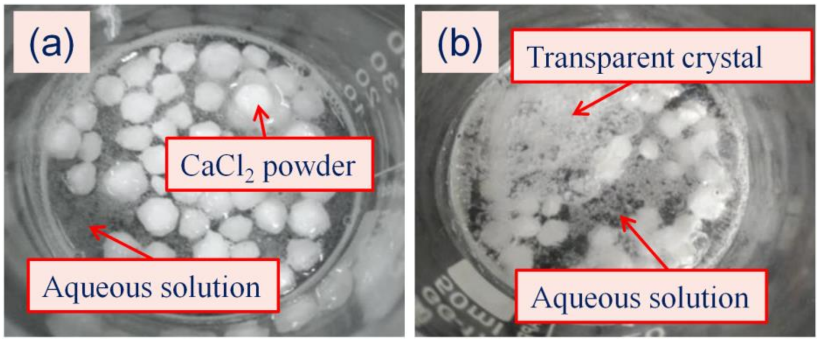

Figure S16. Digital photos of saturated calcium chloride solution before (a) and after (b) 8 hours of heating at 90 °C.

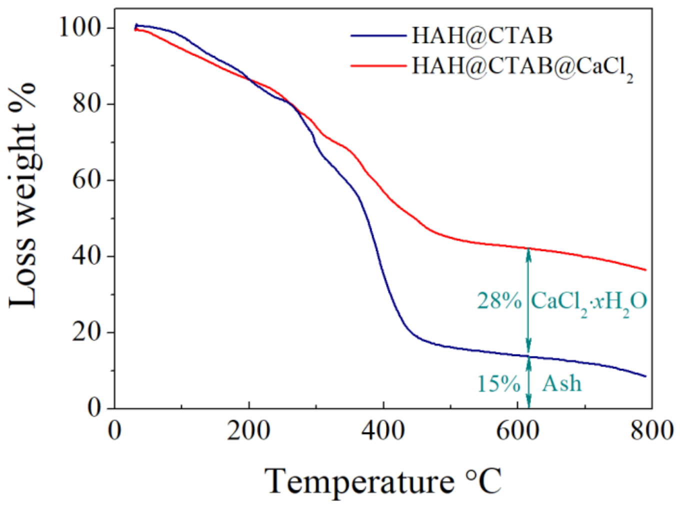

Figure S17. TG curves of the anhydrous hydrogels.
